# Progress in Surgery

**Published:** 1897-09-04

**Authors:** 


### PROGRESS IN SURGERY.

### ABDOMINAL SURGERY.

**Perforating Typhoid Ulcers.**—It is only recently that operation has availed in these terribly fatal complications of typhoid fever, but, considering the circumstances, the successes are very gratifying. Dr. Armstrong (Montreal) has studied the subject, and given the benefit of his researches in a clinical lecture[1]. About 6 per cent. of fatalities in typhoid are due to perforation of the bowel, mostly at the end of the third week, and usually in the ilium within 24 inches of the cæcum. Without operation recovery is very rare indeed, and then only after the formation of a localised abscess.

Laparatomy was suggested by Leyden (Berlin) in 1884, and was first carried out on definite lines by Lücke in 1887. Dr. Armstrong found four successful cases recorded, including one under Van Hook in 1891, the cases of Netschagan and Abbe in 1894, the first-named being done by resection of the bowel and anastomosis. Dr. Armstrong's own case, in 1896, was living 20 days afterwards, and promised well. To these must be added Mr. Bowlby's, and later cases.

The great hindrance to success is the state of the patient; as a rule the operative closure of the ulcer is perfect. Dr. Lauder Brunton and Mr. Bowlby communicated a case to the Royal Medico-Chirurgical Society which occurred in 1895.[2] Perforation took place during convalescence, and laparotomy was performed eighteen hours after acute abdominal pain. The case reads like one of localised abscess which had suddenly given way, and the fact that the operation was not done during the high fever favoured the result. Before the same society, and at the same time, Dr. Herringham and Mr. Bowlby[3] described a case which presented symptoms of perforation; laparotomy was done, but there was no ulcer and no peritonitis; the patient recovered, and the symptoms were referred to constipation. Differences of opinion were expressed as to the tendency for typhoid perforations to be shut off by adhesions. Price reports three cases of typhoid perforation which recovered after suture.[4] In the "Annals of Surgery," March, 1897, Dr. John Finney (Baltimore) gives an elaborate paper based on fifty-two collected cases, which is well worth perusal. About thirteen recoveries up to the present (March, 1897) are recorded, and doubtful cases are discussed.

Attention is called in the paper to the diagnostic value in perforation of an immediate and considerable increase in the number of white corpuscles in the blood. It is not common to find Gaffky's bacillus in the peritoneal fluid. At the end of the paper Dr. Finney tabulates and extracts the cases published up to date.

**Acute Intussusception.**—This affection has nowadays the advantage of earlier surgical treatment than formerly, with corresponding diminution in the mortality of the disease. Dr. Crawford Renton describes three successful cœliotomies for intussusception in children of ten, eleven, and three months respectively.[5] Two successful cases were recently recorded by Mr. Lawford Knaggs[6], one of which had considerable prolapse afterwards at an artificial anus, which was observed to reduce itself spontaneously, "the outer layer being continually inverted at the apex until the whole had disappeared." At the Pathological Society in March, 1897, Mr. D'Arcy Power described the minute anatomy of chronic intussusception; the villi are degenerated, the various coats of the bowel are sclerosed, and are to a large extent indistinguishable from one another, consisting of dense fibrous tissue like an old scar. In the case of spontaneous recovery by separation of the intussuscepted bowel, it was remarked that tryptic digestion is probably a factor. In February, 1897, Mr. D'Arcy Power gave three lectures at the Royal College of Surgeons on intussusception, embracing what is known of the affection and its treatment, as well as the results of his own experiments. He reiterated the futility of injections into the bowel in the enteric form of intussusception; but, unlike some surgeons, he usually precedes abdominal section by attempts at reduction by injections under slight hydrostatic pressure.

**Chronic Intussusception.**—An example of this disease, due to an adenoma of the intestine, was cured by Mr. Wm. Mayo[7] by resection of the bowel, and end-to-end anastosomis with Murphy's button. A similar case was treated successfully and in the same way by Mr. Greig Smith.[8]

**Acute Intestinal Obstruction.**—Mr. M'Ardle[9] has tabulated the results of 41 operations for acute intestinal obstruction, with seven deaths, all patients whose lives were in imminent danger. He quotes that some 94 per cent. of cases of acute intestinal obstruction are worthy of surgical treatment, which to be successful should be performed when there is not excessive collapse. Thus nearly all cases of acute intestinal obstruction should be placed at once under the surgeon, and not treated by enemata, purgatives, and intestinal drugs.

The *Scottish Medical and Surgical Journal*[10] records some exceptional cases under the care of T. Annandale: two strangulated obturator herniæ, a case of obstruction from intestinal concretions, a similar condition with ulceration and acute peritonitis, and a case of fæcal obstruction. One of the cases of strangulated obturator hernia could only be diagnosed after abdominal section, when the knuckle of bowel was found to be gangrenous. The other case was complicated by malignant disease of the rectum, to which the symptoms were attributed, erroneously so as it turned out.

Mr. Lupton[11] describes a case of intestinal obstruction due to four bands, multiplicity being very unusual.

The conditions which may simulate organic obstruction of the large intestine have been summarised by Dr. Thomas Manley (New York).[12] He deals with hæmorrhoidal and atrophic changes in the rectum, and speaks of a large rectal enterolith which he attempted to remove with obstetric forceps! Of other causes of fæcal impaction, Dr. Manley gives enlarged prostate in elderly men as a prolific source of fæcal impaction, and adds visceral displacements, *e g.*, retroflexed uterus, herniæ, mechanical obstruction by neoplasms, and cæcal obstruction to his list of causes of intestinal obstruction other than organic lesions of the bowel itself.

Post-operative intestinal obstruction has always to be borne in mind in abdominal surgery. Boise[13] states that it may be due to one of the four following causes: tonic muscular spasm, true intestinal paralysis, formation of new adhesions, and constrictions undiscovered and unrelieved by the operation. For tonic muscular spasm, which occurs within the first twenty-four hours after operation, nothing is equal to opium or, preferably, codeine.

Heidenhain[14] reports a case of acute intestinal obstruction following resection of the cæcum and ascending colon during convalescence. It was found to be due to a volvulus of the ilium, which was treated by short circuit anastomosis with Murphy's button. The button was later on discharged through a fistula.

**Chronic Intestinal Obstruction.**—Mr. H. H. Clutton[15] has treated a patient with chronic obstruction by resection of the sigmoid flexure. She had had frequent attacks of obstruction for five years previously, which were relieved by enemata, but with increasing difficulty. Murphy's button was used in the operation, which was perfectly successful but for the fact that the button remained *in situ*. This in turn caused obstruction, and Mr. Clutton had to remove it by enterotomy; subsequently peritonitis supervened. The post-operative obstruction occurred in the site of the Murphy button, which had itself travelled backwards in the bowel. Though approving of the button in lateral anastomosis, Mr. Clutton said he would not himself again use the button in end-to-end anastomosis.

Colloid cancer of the cæcum has been treated by excision under the care of Dr. Rolleston and Mr. Marmaduke Sheild.[16] The mesentery was freely removed and the ileum and colon united by end-to-end sutures. Drainage was provided and a little fæcal leakage occurred on the twelfth day, but the patient, who was twenty-nine years of age, recovered.

[1] Brit. Med. Journ., December 5, 1896. [2] Brit. Med. Journ., Jan. 30, 1897. [3] Brit. Med. Journ., Jan. 30, 1897. [4] Philadelphia Policlinic, Nov. 14, 1896. [5] Brit. Med. Journ., Oct. 17, 1896. [6] Lancet, April 24, 1897. [7] Annals of Surgery, Dec., 1896. [8] Lancet, Jan. 4, 1896. [9] Dublin Journ. Med. Science, April 1, 1897. [10] Scottish Med. and Surg. Journ., Feb., 1897. [11] Lancet, May 1, 1897. [12] New York Med. Record, Mar., 1897. [13] Med. News, July 18, 1896. [14] Centralbl. für. Chirurgie, No. 49, 1896. [15] Brit. Med. Journ., Oct, 3, 1896. [16] Lancet, Dec. 19, 1896.

### GENERAL SURGERY.

**Antiseptics.**—The local ill-effects which follow the use of iodoform are described by Dr. J. Tussau,[1] under the name of surgical iodoformism, in contradistinction to the manifestations of acute iodism or intoxication, the result of absorption. The latter belongs to the domain of toxicology, and has been thoroughly described by Brun, Audry, Iscovesco, and others. According to Tussau surgical iodoformism comprises three stages—(1) After a longer or shorter period of toleration, the wound, while secreting no pus, is surrounded by an inflammatory areole. Sometimes there developes at its circumference a series of vesicles, a condition to which he has given the name of iodoformic herpes. During this stage also petechiæ occasionally appear locally or at a distance, disseminated or grouped in patches. The wound stagnates, and becomes inflamed, instead of progressing towards healing. (2) These symptoms, having existed for a variable period, are followed by itching between the fingers (for the fingers and hands are the most sensitive to iodoform), generalised pruritus, vesicles along the paths of the collateral nerves of the fingers (iodoformic zoster), and later on by blebs and diffuse phlyctenæ. Areolar or pseudo-erysipelatous lymphangitis makes its appearance in the limb affected by the iodoform. (3) If the use of iodoform is persisted in this lymphangitis progresses, the tongue becomes coated, and the patient becomes agitated and sleepless. A genuine phlegmonous state, with general reaction, developes. The cellular tissue may mortify, and necrosis of the connective, cartilaginous, or osseous tissue may threaten the patient with loss of a limb or even his life. The use of iodoform for a dressing should immediately cease, and the patient should be informed of his susceptibility to this substance. The replacement of antisepsis by asepsis has lessened the field of usefulness of iodoform, the principal indication for which is in the case of tuberculous abscesses. Dr. Carl S. Hægler[2] finds that the delay in the growth of organisms produced by airol is slightly greater than that resulting from iodoform, and infinitely more than the effect of dermatol. He found that the influence of antiseptic powders is greater the earlier their use is commenced; in acute phlegmonous processes they do but little good, while the more chronic the inflammation the better the results obtained, whence their special indication in tuberculosis. For injecting tuberculous joints Hægler uses a 10 per cent. emulsion of airol. He has employed airol in over 2,000 cases. Zeroform, or Bismuth tribromophenyllicum, as a surgical antiseptic, has been further investigated by C. G. Cumston.[3] It appears to have a soothing influence on burns, like iodoform. After the curettement of tuberculous abscesses or glands, its application is followed by rapid cicatrisation. On account of the continual development of tribromophenol and oxide of bismuth a wound will be left in a perfectly aseptic condition, while the slightly irritating action of the former gives a fresh and healthy aspect to the wound. In septic and tuberculous sores after failure of iodoform, scraping, &c., A. G. Miller[4] advises a resort to sulphur. The action of sulphur may be mitigated by mixing it with glycerine (ʒj to ℥j); such a mixture, with the addition perhaps of some carbolic lotion, makes a good injection for septic or tuberculous cavities. In the case of an ulcer, the sulphur, in fine powder, should be gently rubbed in with the finger, and the wound or sore dressed with an antiseptic dressing. Twenty-four hours is sufficient time for the sulphur to produce its destructive action in a recent wound, but in septic and tuberculous sores a longer period is necessary. Formalin-gelatin has been used by Thomalla[5] for clean incisions, and has produced primary union. When used as a dressing to ragged and irregular wounds, suppuration does not occur. Necrotic fragments quickly separate, and cicatrisation takes place more rapidly than under other dressings. Formalin-gelatin is especially serviceable in the treatment of burns. Originally Thomalla removed the scabs, but as suppuration never took place he allowed them to remain, and has never had infection, excepting in one case. This was a burn of the foot, which was prevented from healing by the rubbing of the shoe.

Amyliform is believed by Classen[6] to be a true chemical combination of starch and formaldehyde. He states that it is absolutely free from irritating properties, and non-toxic; that it favourably affects the secretion and prevents tissue necrosis; that it does not form a dry crust which retains secretion; and that it will absolutely prevent the foul odour from gangrenous wounds. Dr. O. Kawalewsky[7] has attempted to ascertain whether the action of some antiseptics, viz., iodine, iodide of potassium, bichloride of mercury, and zinc chloride, was due to their bactericidal properties or to their producing a leucocytosis. He concludes that these antiseptics act equally as much through chemotaxis, thus causing leucocytosis, as by their bactericidal properties. Dr. Gueorguiwsky,[8] a Russian army physician, has made the important observation that compresses, soaked in a 2 per cent. solution of chemically pure sodium bicarbonate, covered with some impermeable tissue, will stop purulent secretion and phlegmonous inflammation more rapidly than all ordinary antiseptics, such as carbolic acid, iodoform, &c. In every instance of whitlow or diffuse phlegmonous inflammation, the pain and the suppuration were arrested, and cure was rapidly completed without the use of any drainage, which is so often the cause of intense suffering to the patient. Silver and silver salts are advocated by Credé[9] as safe antiseptic agents in the treatment of wounds. When gauze impregnated with finely divided silver is used for ulcerated or wounded parts which secrete freely, the silver is attacked by the products of decomposition and is converted into a lactate of this metal, which is very irritating. Open surfaces of some standing are therefore covered at first by powdered citrate of silver (itrol), and then excluded from the air by the silver gauze. This method of treatment, it is stated, does not need such strict precautions as other methods of antiseptic treatment. A. G. Miller[10] is an advocate for dry dressings, thinking that starving microbes is as good a way of killing them as poisoning them. His methods are as follows: In an ordinary aseptic operation, having used antiseptic lotions to make hands, instruments, and the part to be operated on as aseptic as possible, he thoroughly dries the operation area of skin and then washes the skin with ether. Towels wrung out of carbolic lotion, or warm and dry from a steriliser, surround the operation area. Swabs or sponges to be used for drying the wound are wrung out of carbolic lotion, and made as dry as possible. No lotion is splashed or irrigated over the wound. The hands of the operator and assistants are occasionally dipped in some antiseptic lotion, but shaken dry or wiped on a swab. At the conclusion of the operation the entire raw surface is dusted with a powder (boracic acid 7 and iodoform 1), which is well rubbed in with a damp swab. After the stitches are inserted the wound and surrounding parts are dusted with the iodoform and boracic powder, a layer or two of iodoform gauze applied, and over that plenty of wool and a bandage. If oozing is expected wood wool is employed. Drainage tubes are seldom required. Recently, Mr. Geo. Stoker[11] has recorded the results of his new treatment by oxygen gas of chronic ulcers, wounds, burns, and other diseases as lupus. After trying antiseptic vapour, such as carbolic acid, thymol, &c., he has discarded all antiseptics in favour of oxygen, which possesses great powers of destroying bacteria in a wound, of relieving pain, preventing the formation of fœtid discharges, and of causing rapid healing. Although a number of cases of septic trouble have been reported as being treated by anti-streptococcic serum with varying results, it seemed to W. Watson Cheyne[12] that the use of this serum should not be confined to cases in which streptococcal infection has already occurred, but that its main utility will be as a prophylactic in cases where infection is likely to occur, more especially in cases of operations on the tongue and throat and about the rectum. In operations about the pharynx, the tonsil, and base of tongue septic pneumonia is a very frequent cause of death. Cheyne relates three cases: (1) a case of carcinoma of base of tongue involving the anterior pillar of fauces and tongue; (2) an extensive malignant growth of left side of lower jaw, spreading to the cheek, fauces, and upper jaw; (3) an extensive epithelioma of tongue, spreading to tonsil and floor of mouth, in which this plan was carried out, and they certainly, he says, "followed a course which he had not previously experienced in these most extensive operations. If by this means one can prevent the occurrence of septic pneumonia and the diffuse septic process which are so apt to follow these extensive operations, a great step in advance will have been made as regards the feasibility of carrying them out." G. L. Cheatle[13] reports six cases in which a virulent streptococcus pyogenes was found. Three of these were treated by the anti-streptococci serum as well as by operative and antiseptic surgical methods. One case was treated by the serum alone; but two were treated by operative and antiseptic surgical procedures alone, which prove that properly-conducted surgical means can cure even when the patient suffers from a severe streptococcus injection, whether that injection is pure or mixed. Therefore, when a case is treated surgically as well as by the injection of the anti-toxin, it must not be too hastily assumed that the latter was the cause of the cure.

[1] Medical Week, Nov. 13, 1896, p. 542. [2] Beiträge zur Klinische Chirurgie, xv. 1, s. 266; Epit. Brit. Med. Journal, April 24, 1897; and Annals of Surgery, March, 1897, p. 371. [3] Boston Med. and Surg. Jour., Jan. 14, 1897; and Epit. Brit. Med. Jour., March 6, 1897. [4] Practitioner, Feb., 1897, p. 160. [5] Therapeutische Monatshefte, Jan., 1897; and Therapeutic Gazette, March 15, 1897, p. 196. [6] Ibid., p. 197. [7] Inaugural Dissertation, Berne; and Annals of Surgery, March, 1897, p. 370. [8] Medical Week, March 19, 1897. [9] Centralbl. f. Chirurg., No. 43, 1896; and Epit. Brit. Med. Journal, Mar. 20, 1897, p. 46. [10] Practitioner, Jan., 1897, p. 28. [11] Lancet, Feb. 13, 1897; and Guy's Hospital Gazette, March 13, 1897, p. 155. [12] Practitioner, April, 1897, p. 347. [13] Lancet, Jan. 2, 1897, p. 24.

